# Impact of highway traffic and the acoustic screen on the content and spatial distribution of heavy metals in soils

**DOI:** 10.1007/s11356-017-8910-z

**Published:** 2017-03-30

**Authors:** Szymon Różański, Hanna Jaworska, Katarzyna Matuszczak, Joanna Nowak, Amber Hardy

**Affiliations:** 1 Department of Soil Science and Soil Protection, Faculty of Agriculture and Biotechnology, UTP University of Science and Technology, Bernardynska St.6, 85-029 Bydgoszcz, Poland; 2 Department of Natural Resources Science, Laboratory of Pedology and Soil Environmental Science, University of Rhode Island, Kingston, RI USA

**Keywords:** Heavy metals in soils, Highway traffic pollution, Geo-accumulation index

## Abstract

Recent years have witnessed intensification of road traffic and, with it, the amount of substances emitted by vehicles. Such emissions need to be monitored for public health purposes. The aim of this study was to evaluate the impact of the highway traffic on the total content and bioavailability of Zn, Cu, Ni, Cd, Cr and Pb in nearby soils as well as influence of an acoustic screen on spatial distribution of the metals. The material included 40 soil samples collected from 15 research points located 5, 10, 25 and 50 m away from the road acoustic screen and from 4 points between the screen and the highway. Additionally, 5 research points were located next to the metal barrier. Selected physicochemical properties of soils were determined: soil texture, soil pH, TOC and CaCO_3_ content. The total content of heavy metals in the soils was determined by AAS after digestion in *aqua regia* and bioavailable forms in 1 M diethylenetriaminepentaacetic acid. The research found low impact of the highway traffic on the content of heavy metals in soils; however, due to a very short period of this potential impact (5 years), the moderately polluted category of geo-accumulation index of cadmium and high bioavailability of lead indicate the need of repeating the research within the next several years. Furthermore, the road acoustic screen significantly influenced spatial distribution of the metals in soils.

## Introduction

The rise of mechanized transportation is associated with the development of road infrastructure. Road traffic is a major source of substances in the soil and atmosphere (Pallavi and Harrison 2013), including, among others, heavy metal compounds (Johanssona et al. 2009). The content of certain compounds in the soil is directly proportional to vehicle speed. Higher vehicle velocity causes increased emissions of exhaust containing, among other compounds, heavy metals (Duong and Lee 2011). Pollutant emissions must be monitored to preserve environmental quality and prevent its degradation (Franco et al. 2013).

In Poland, many highways are located along farmlands (Lewin et al. 2015). Pollution emitted by vehicles may be transported to these fields. There is a high risk of direct contamination of crops by heavy metals (Gill et al. 2014). The content of heavy metals in soil may be affected by natural (climate, soil processes) and anthropogenic factors such as agriculture and road traffic (Wei et al. 2007). Many studies have focused on total emission of heavy metals into agricultural areas (Hjortenkrans et al. 2006). The content of heavy metals in soils located along roads is strongly related to traffic and decreases with increasing distance from the road (Temmerman et al. 2003).

Uptake of heavy metals from soil by plants may be either passive or active (Kim et al. 2003). The main source of heavy metals in plants is road traffic (Gworek et al. 2011). Dynamic development of motorways and roads causes increasing content of heavy metals in many components of the environment (Blagojević et al. 2009). Automobiles, especially those used for freight, constitute an important source of heavy metals, especially lead, nickel and cadmium (Falahi-Ardakani 1984).

Heavy metals occurring in bedrock are mostly in stable forms and slowly undergo mobilisation during weathering processes (Karczewska and Kabała 2010). Soil pH, organic carbon content and clay content significantly impact the accumulation of heavy metals in soil (Malczyk and Kędzia 1996; Rosada 2007). The toxic effect of heavy metal pollution in soil is not just the result of the total content but also the chemical form of the element (Kabata-Pendias 2011). Diethylenetriaminepentaacetic acid (DTPA) is a common reagent used to determine the content of potentially bioavailable forms of metals in soils (Maiz et al. 2000; Dai et al. 2004; Feng et al. 2005). These forms constituted part of the total content (Elsokkary 1978), but many authors state these results are overestimated (McGrath 1996; Sterckeman et al. 1996). Heavy metals in the soil from anthropogenic sources tend to be more mobile than forms of pedogenic or lithogenic origin (Wuana and Okieimen 2011).

The aim of this study was to evaluate the impact of traffic in the vicinity of an acoustic screen on the spatial distribution of zinc (Zn), copper (Cu), nickel (Ni), cadmium (Cd), chromium (Cr) and lead (Pb) in soils intensively cultivated for food production after 5 years of highway running. It is widely known that concentration of heavy metals is the highest in soils close to roads and decreases with distance. It was assumed that the investigated screen, besides its acoustic role, also blocks wind. It is solid and therefore disturbs airflow, which may cause unpredictable impacts on the distribution of highway traffic contaminants in the analysed soils. Bioavailability of the metals in relation to the soil parameters was also assessed.

## Materials and methods

### Description of the study area and soil sampling

Research material was collected from soils along the A1 Grudziądz Highway (Poland) (53° 21′ 14.6″ N 18° 42′ 13.1″ E). The investigated area was covered by Luvisols (IUSS 2014) formed from glacial till (Uniejewska 1982). The highway has been in use since 14 October 2011. The traffic intensity is over 17,000 vehicles per day (General Directorate 2015). There was an intensive small-scale cereal-livestock system with wheat in the year of sampling (in crop rotation with sugar beet and maize). Wind direction in the region is varied, with about 35% from the west, 25% from the east, 20% from the north and 20% from the south throughout the year (Woś 2010). Road acoustic screens are common constructions in Poland, protecting single houses, settlements or even cities in close vicinity of roads from noise. These barriers are usually made of steel, but also concrete or plastic. They are long—several dozen meters to many kilometres. The investigated screen was made of steel plates, about 650 m long and 4 m high.

**Fig. 1:**
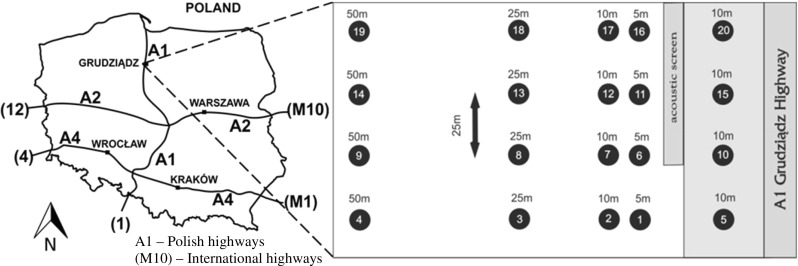
Location of research points

The material included 40 soil samples collected from 15 research points located 5, 10, 25 and 50 m away from a road acoustic screen and from 4 points between the screen and the highway. Furthermore, 5 sampling points were located next to the metal barrier (Fig. 1). The distance between the points was 25 m. Samples were taken using a hand auger from two depths: 0–20 cm (surface samples) and 20–40 cm (subsurface samples). The bucket was advanced to the appropriate depth and the contents (4 subsamples from each point) were transferred to a homogenization container for processing. The material was collected in July 2016 during 1 day of outdoor work.

### Laboratory and statistical analysis

In the air-dried soil samples, crushed and passed through a 2-mm sieve, the following analysis was conducted: pH using a glass electrode in a 1:5 (by volume) suspension of soil in deionized water (pH in H_2_O) and again in 1 M potassium chloride solution (pH in KCl) (ISO 10390: 2005), total organic carbon content (TOC) by sulfochromic wet oxidation method in the potassium dichromate solution—K_2_Cr_2_O_7_ (ISO 14235: 1998), carbonate content in calcimeter according to the volumetric Scheibler’s method and soil texture by sieving and sedimentation method (ISO 11277: 2009). The total content of heavy metals was determined using AAS method (SOLAR S4) after digestion in 3:1 HCl and HNO_3_ acids (*aqua regia*) (ISO 11466: 1995) and bioavailable forms after extraction with 1 M DTPA (Lindsay and Norvell 1978). The methods used are common in soil science laboratories. All analyses were conducted in three replicates and the validation of the results was based on the certified materials (reference soil sample TILL-3 and SO-4; Canada Centre for Mineral and Energy Technology). Pearson’s correlation coefficient (*p* < 0.05) was used to explore relationships between the total content of analysed metals, their available forms and soil parameters, as well as ANOVA analysis for the total content of metals in surface horizon with the distance from the acoustic screen. Statistical analysis of the results was calculated in the Statistica 12.0 for Windows Pl software.

### Pollution index

Geo-accumulation index (Igeo) (Müller 1969) makes an assessment of the impact of heavy metals on the soil environment (Saeedi et al. 2012; García-Martínez and Poleto 2014). Igeo (Müller 1969) was calculated as follows:

1 $$ \mathrm{Igeo}= \log \left(\frac{C\mathrm{s}}{1.5\ \mathrm{x}\  B\mathrm{n}}\right) $$

where *C*s refers to calculated values and *B*n to background values (Czarnowska 1996), the factor 1.5 is applied in order to control the variations of *B*n values caused by the environment. Shi et al. (2010) and García-Martínez and Poleto (2014) identified seven categories of Igeo, shown in Table 1.

**Table 1:** Geo-accumulation index (Müller 1969)

Value	Category
Igeo ≤0	Unpolluted
0 <Igeo <1	Unpolluted to moderately polluted
1 <Igeo <2	Moderately polluted
2 <Igeo <3	Moderately to highly polluted
3 <Igeo <4	Highly polluted
4 <Igeo <5	Highly to extremely polluted
Igeo ≥5	Extremely polluted

Applied geochemical background values were from Czarnowska (1996), for glacial till as a parent material, of analysed soils as follows: 33.0 mg kg^−1^ (Zn), 8.30 mg kg^−1^ (Cu), 11.0 mg kg^−1^ (Ni), 0.19 mg kg^−1^ (Cd), 26.0 mg kg^−1^ (Cr) and 10.3 mg kg^−1^ (Pb).

## Results

The analysis of texture allowed classification of the investigated soils into 2 texture classes: sandy loam and sandy clay loam (USDA 2012). The percentage of sand ranged from 57.0% to 72.0%, silt from 16.0% to 26.0% and clay from 7.0% to 21.0%. The content of total organic carbon (TOC) ranged from 1.8 to 10.7 g kg^−1^ in surface samples and from 0.9 to 9.3 g kg^−1^ in subsurface samples. In the analysed samples pH_H2O_ ranged from 6.0 to 8.0, while pH_KCl_ from 5.3 to 7.4. The content of CaCO_3_ ranged from <1 to 6.4% (Table 2).

**Table 2:** Physicochemical properties of soil samples

Soil sample and depth (cm)		pH_H2O_	pH_KCl_	TOC (g kg^−1^)	CaCO_3_ (%)	Clay (%)
1	0–20	7.6	7.2	3.8	<1	10.0
	20–40	7.6	7.2	2.6	<1	11.0
2	0–20	7.0	6.5	8.6	<1	10.0
	20–40	7.3	6.8	5.4	<1	10.0
3	0–20	7.1	6.5	4.2	<1	11.0
	20–40	7.0	6.3	2.7	<1	10.0
4	0–20	7.1	6.5	2.7	<1	17.0
	20–40	7.2	6.8	9.3	<1	9.0
5	0–20	7.4	7.1	9.3	1.3	14.0
	20–40	7.5	7.2	5.6	1.4	16.0
6	0–20	7.4	7.1	3.9	<1	21.0
	20–40	7.1	6.4	2.0	<1	19.0
7	0–20	7.4	7.0	2.9	<1	7.0
	20–40	7.1	6.3	1.9	<1	15.0
8	0–20	7.3	6.9	2.1	<1	9.0
	20–40	7.2	6.8	3.2	<1	12.0
9	0–20	7.6	7.2	1.8	4,6	14.0
	20–40	7.7	7.3	1.5	5.3	17.0
10	0–20	7.6	7.3	4.6	1.4	13.0
	20–40	8.0	7.4	0.9	5.6	12.0
11	0–20	7.2	6.9	5.5	<1	10.0
	20–40	7.1	6.6	4.0	<1	9.0
12	0–20	7.3	7.1	7.7	<1	7.0
	20–40	7.3	7.0	6.5	<1	8.0
13	0–20	7.5	7.1	6.3	1.3	12.0
	20–40	7.5	7.2	7.6	1.5	10.0
14	0–20	7.3	6.8	4.6	<1	12.0
	20–40	7.2	6.8	4.9	<1	10.0
15	0–20	7.8	7.3	1.9	2.3	13.0
	20–40	7.7	7.3	1.5	2.8	12.0
16	0–20	7.1	6.8	4.3	<1	8.0
	20–40	7.2	6.8	5.7	<1	9.0
17	0–20	6.0	5.3	6.9	<1	10.0
	20–40	6.4	5.6	5.6	<1	9.0
18	0–20	7.4	7.2	4.9	1.3	13.0
	20–40	7.7	7.3	6.2	3.9	15.0
19	0–20	6.9	6.8	10.7	<1	19.0
	20–40	7.4	6.6	3.9	<1	15.0
20	0–20	7.6	7.2	3.6	2.0	11.0
	20–40	7.9	7.3	2.9	6.4	17.0

The highest total content of heavy metals in analysed soils was seen in zinc (18.40–46.53 mg kg^−1^) and chromium (15.98–31.88 mg kg^−1^). The lowest total content was in lead (0.00–4.55 mg kg^−1^) and cadmium (0.00–1.25 mg kg^−1^) (Table 3).

**Table 3:** The total content and bioavailable forms of heavy metals in soil samples (mg kg^−1^)

Soil sample and depth (cm)		Zn		Cu		Ni		Cd		Cr		Pb	
		Total	DTPA	Total	DTPA	Total	DTPA	Total	DTPA	Total	DTPA	Total	DTPA
1	0–20	34.25	2.03	5.70	0.75	6.98	0.35	0.33	b.d.l.	19.68	1.16	1.55	0.39
	20–40	34.88	6.98	6.10	0.88	5.93	0.31	0.25	b.d.l.	22.50	0.13	2.53	0.72
2	0–20	29.03	2.22	6.00	0.79	3.40	0.20	0.90	b.d.l.	20.15	0.84	1.30	0.18
	20–40	23.43	3.10	6.58	0.83	6.40	0.32	0.30	b.d.l.	21.45	1.00	2.10	0.38
3	0–20	20.25	2.57	7.20	1.14	5.85	0.33	0.53	0.03	17.85	0.66	3.75	0.36
	20–40	19.80	4.23	7.48	1.03	10.18	0.50	1.18	b.d.l.	15.98	0.28	2.85	0.23
4	0–20	20.23	1.55	8.13	0.62	5.65	0.29	1.03	b.d.l.	18.58	0.41	1.55	0.42
	20–40	18.40	3.11	8.28	0.77	7.05	0.33	1.18	b.d.l.	19.78	0.52	1.68	0.67
5	0–20	25.48	1.81	8.88	0.69	8.85	0.22	1.03	b.d.l.	22.13	0.48	2.28	0.38
	20–40	27.30	1.01	9.78	0.80	9.80	0.18	0.70	0.03	21.58	0.79	3.08	0.66
6	0–20	31.28	0.56	4.85	0.43	13.28	b.d.l.	b.d.l.	0.20	26.85	0.79	b.d.l.	b.d.l.
	20–40	30.73	0.57	5.50	0.36	11.28	0.17	0.10	0.19	26.60	0.99	b.d.l.	b.d.l.
7	0–20	30.08	1.13	5.13	0.59	13.13	0.24	0.10	0.16	30.35	0.45	b.d.l.	b.d.l.
	20–40	32.20	0.87	5.15	0.51	9.60	0.20	0.12	0.19	22.13	0.80	b.d.l.	b.d.l.
8	0–20	25.55	0.76	4.38	0.39	4.33	0.05	b.d.l.	0.13	19.25	0.72	b.d.l.	b.d.l.
	20–40	26.23	0.59	4.58	0.43	8.58	0.06	b.d.l.	0.13	20.88	1.05	0.88	0.58
9	0–20	27.13	0.86	5.83	0.56	11.38	0.31	b.d.l.	0.07	31.88	0.76	b.d.l.	b.d.l.
	20–40	23.85	14.37	4.98	0.57	9.33	0.05	0.38	0.07	23.90	1.12	b.d.l.	b.d.l.
10	0–20	25.25	12.04	5.70	0.63	6.50	0.16	b.d.l.	b.d.l.	26.65	0.47	b.d.l.	b.d.l.
	20–40	24.65	2.76	5.85	0.64	7.73	b.d.l.	0.18	b.d.l.	29.43	0.26	b.d.l.	b.d.l.
11	0–20	24.28	14.20	6.63	0.50	7.83	0.22	0.18	b.d.l.	26.18	0.61	0.25	0.11
	20–40	24.88	12.24	6.93	0.62	3.40	0.15	b.d.l.	0.04	19.68	0.98	1.13	0.88
12	0–20	28.48	16.68	4.10	0.44	7.18	0.12	b.d.l.	0.06	27.45	0.45	1.60	1.02
	20–40	26.10	12.15	7.63	0.52	7.93	0.06	0.08	0.05	27.43	0.32	1.35	0.99
13	0–20	37.05	13.45	9.15	0.53	8.30	0.16	b.d.l.	b.d.l.	30.25	0.56	1.55	1.14
	20–40	36.78	14.90	8.30	0.50	5.18	0.65	0.85	b.d.l.	25.48	1.06	3.03	1.58
14	0–20	24.73	1.18	6.65	0.75	6.13	0.20	0.75	b.d.l.	24.38	0.73	1.80	0.90
	20–40	21.38	15.78	7.80	0.80	5.95	0.13	0.50	b.d.l.	24.18	0.35	2.70	0.88
15	0–20	22.60	1.56	7.73	0.85	7.45	0.24	0.63	b.d.l.	27.00	0.84	0.55	0.42
	20–40	24.23	0.88	8.18	0.74	4.15	0.33	1.25	b.d.l.	23.33	1.09	4.38	0.96
16	0–20	42.30	15.94	3.25	1.11	6.70	0.25	b.d.l.	b.d.l.	16.38	0.67	4.55	b.d.l.
	20–40	24.30	18.76	2.88	1.43	9.33	0.56	b.d.l.	b.d.l.	19.63	0.45	3.95	0.35
17	0–20	27.85	1.59	2.90	0.92	8.10	0.29	b.d.l.	b.d.l.	19.88	0.84	2.53	0.46
	20–40	26.88	1.46	3.93	0.78	4.80	0.16	0.15	b.d.l.	19.05	0.76	3.10	0.52
18	0–20	34.50	8.94	5.00	0.69	8.45	0.42	0.10	b.d.l.	20.78	0.31	0.43	0.41
	20–40	41.38	2.25	6.05	0.68	8.95	0.44	0.15	b.d.l.	24.75	0.50	1.68	0.81
19	0–20	46.53	1.24	6.33	0.67	9.90	0.46	b.d.l.	b.d.l.	20.25	0.20	2.35	0.38
	20–40	41.28	1.06	7.38	0.68	11.95	0.31	0.53	b.d.l.	26.43	0.56	2.28	0.65
20	0–20	36.08	4.34	5.38	0.86	8.85	0.27	0.40	b.d.l.	21.50	0.76	2.48	0.40
	20–40	37.98	1.91	8.10	0.73	9.88	0.06	0.45	b.d.l.	21.45	0.85	3.95	0.11
Mean	0–20	29.65	5.23	5.95	0.70	7.91	0.24	0.30	0.03	23.37	0.64	1.43	0.35
	20–40	28.33	5.95	6.57	0.72	7.87	0.25	0.42	0.04	22.78	0.69	2.03	0.55
Min	0–20	20.23	0.56	2.90	0.39	3.40	b.d.l.	b.d.l.	b.d.l.	16.38	0.20	b.d.l.	b.d.l.
	20–40	18.40	0.57	2.88	0.36	3.40	b.d.l.	b.d.l.	b.d.l.	15.98	0.13	b.d.l.	b.d.l.
Max	0–20	46.53	16.68	9.15	1.14	13.28	0.46	1.03	0.20	31.88	1.16	4.55	1.14
	20–40	41.38	18.76	9.78	1.43	11.95	0.65	1.25	0.19	29.43	1.12	4.38	1.58
SD	0–20	7.01	5.82	1.70	0.21	2.57	0.11	0.38	0.06	4.63	0.22	1.31	0.34
	20–40	6.89	6.19	1.73	0.23	2.42	0.18	0.41	0.06	3.29	0.32	1.39	0.42

*b.d.l.* below detection limit

The mean percentage of total metals that were in bioavailable forms were: 19.32% (Zn), 11.35% (Cu), 3.10% (Ni), 9.76% (Cd), 2.88% (Cr) and 25.79% (Pb).

**Fig. 2:**
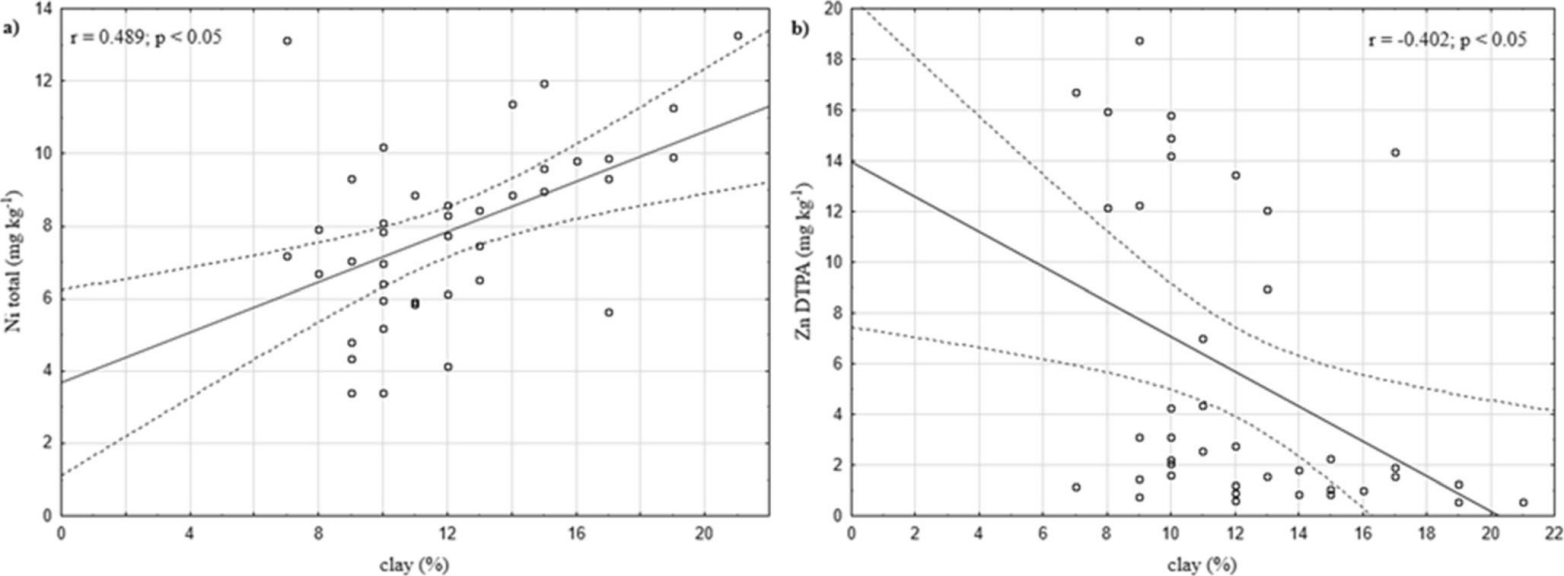
Correlation with a significant relationship between the clay content and the total content of nickel (**a**) and bioavailable form of zinc (**b**)

Calculated correlation coefficients confirmed the significant relation between the content of clay and the total content of nickel (*r* = 0.489; *p* < 0.05) (Fig. 2a) and bioavailable form of zinc (*r* = −0.402; *p* < 0.05) (Fig. 2b).

Significant coefficients calculated in ANOVA for the metal content in surface horizons and distance from the acoustic screen proved the influence of the screen on spatial distribution of the metals (Table 5).

Average geo-accumulation indexes took the following values: zinc −0.234, copper −0.337, nickel −0.347, cadmium 0.292, chromium −0.230 and lead −1.011 (Table 4).

**Table 4:** Calculated geo-accumulation indexes (Igeo)

	Zn	Cu	Ni	Cd	Cr	Pb
Mean	−0.234	−0.337	−0.347	0.292	−0.230	−1.011
Min	−0.389	−0.633	−0.690	−0.212	−0.377	−1.791
Max	−0.027	−0.089	−0.098	0.556	−0.088	−0.531
SD	0.098	0.136	0.143	0.258	0.085	0.345

## Discussion

Soils generally had low total and bioavailable trace element contents. According to Polish guidelines (Regulation 2016), the tested area may qualify as unpolluted by the analysed metals. Moreover, determined values were characterised for uncontaminated soils of Europe (De Vivo et al. 1997; Salminen and Tarvainen 1997; Salminen and Gregorauskienė 2000; Sierra et al. 2007). The content of the metals followed the sequence: Zn > Cr > Ni > Cu > Pb > Cd. High concentrations of chromium (up to 47.49 mg kg^−1^) are typical for soils under agricultural and traffic influence, but then the content of lead is usually much higher (70.36 mg kg^−1^) than in the tested soils (Liu et al. 2013). The lower content of Cr is more characteristic of urban sites under different land uses - Zn > Cu > Pb > Cr (Trujillo-González et al. 2016). Most urban soils have elevated values of cadmium, copper, lead and zinc like soils of Hong Kong (Li et al. 2001), or even exceed the limits, like in soils of Naples (Italy) in the case of lead, cadmium and zinc (Imperato et al. 2003). In the investigated soils, there were almost non﻿e significant correlations between total content of the metals and soil parameters. Only nickel was positively correlated with clay content (*r* = 0.489; *p* < 0.05) (Fig. 2a), which indicates the significant role of clay minerals in bonding of this metal (Kabata-Pendias 2011).

In research conducted in Istanbul close to the E-5 Highway, heavy metals such as Pb, Cu, Mn, Cd and Ni have been detected in street dust (Sezgin et al. 2004). In roadside soils of Damascus, lead concentration ranged from 78.4 to 832.0 mg kg^−1^ (Othman et al. 1997), and in soils along A-8 Highway in Gipuzkoa (Spain), remarkable high levels of zinc and lead were determined (Garcia and Millán 1998). Other research showed that the content of heavy metals in soil samples from local roads was much higher than in samples along highways (Huber et al. 2016). Concentration of pollutants in soils is also affected by traffic volume (Horstmeyer et al. 2016) as well as the speed of vehicles (Duong and Lee 2011). So possibly the emission of exhaust in traffic jams is even higher than during high speed transportation. Other factors like construction of the road, velocity and direction of air flow (wind) or infrastructure along the road may also influence the concentration of pollutants in soils. Undoubtedly, automobiles constitute the main source of heavy metals in roadside soils (Aslam et al. 2013). In comparison to other Polish highways, the investigated road classifies as a medium traffic road, with 17,188 vehicles/day. The mean traffic volume on international Polish roads in 2015 was 20,067 vehicles/day, with a maximum of up to 100,983 vehicles/day (General Directorate 2015). In combination with the relatively short time of contamination source impact, the effect may be imperceptible. However, the comparative research conducted in Poland (Gliwice and Opole), Germany (Tübingen, Ulm and Böblingen), Finland (Helsinki), Tajikistan (Dushanbe) and China (Lanzhou) showed significant increase of Zn, Pb, Cd and Cu content on test-monitoring plots installed next to the road edge for 24 months (Wawer et al. 2015). These amounts depended on place and element and were about 2–50 mg kg^−1^ year^−1^ for Zn, 0–30 mg kg^−1^ year^−1^ for Pb, 0–2 mg kg^−1^ year^−1^ for Cd and 1–24 mg kg^−1^ year^−1^ for Cu. It indicates that the potential accumulation of these pollutants may be noticeable but of course dependent on many other factors responsible for metal bonding (soil properties, form of the metal etc.).

Assessing the metal concentration according to the calculated geo-accumulation index, the investigated soils may be classified from unpolluted to moderately polluted. The higher value of this index was determined for Cd (max. 0.556); in all other cases, negative values were noted (Table 4). Trujillo-González et al. (2016) reported that in the highway soils the geo-accumulation index took the following mean values: Pb 0.8, Ni −2.1, Zn 0.5, Cu 0.9 and Cr 2.7. Another study showed that the mean Igeo index in commercial soils of Shanghai was 2.3 for Pb, 1.5 for Cu and 2.4 for Zn (Shi et al. 2010). This indicates that the geo-accumulation index depends on the type of traffic sectors (commercial, residential or highway). Many studies confirmed that Igeo is higher in commercial sectors than in the highway sectors (Shi et al. 2010; Wei and Yang 2010). So, the obtained index may prove that highway traffic did not have a significant influence on the content of heavy metals in soil. Furthermore, Igeo index showed that the acoustic road screen and distance from the highway also did not impact the content of heavy metals in soils. Moreover, the screen installed along A1 Grudziądz Highway did not stop migration of pollutants. This situation was difficult to assess because the investigated highway has been in use since 14 October 2011, so the period of potential impact is very short. Research conducted by Hofman and Wachowski (2010) on new section of A2 Highway (Poznań) which has been in use since 2003 showed low concentration of lead not exceeding the permissible standards. A different result was obtained by Czarnowska (1994) in soils located along exit roads of Warsaw. This research showed contamination by lead in a 30-m zone away from these roads. A reason for this could be the retraction of leaded gasoline in Poland in February 2005. A similar study demonstrated that traffic had an effect on the accumulation of Zn, Pb, Cd and Cu in soils at least at distances up to 50 m (Czarnowska et al. 2002).

On the basis of ANOVA the relationship between the distance from the acoustic screen and the concentration of analysed metals in surface horizons was confirmed (Table 5). Significant coefficients showed a slight trend in spatial distribution of investigated metals. The highest average concentrations were noticed 5 and 50 m away from the screen. In comparison to standard distribution of traffic contaminants in soils along the roads, where concentration decrease with distance, and reach natural levels 30–50 m away (Czarnowska 1994; Temmerman et al. 2003; Werkenthin et al. 2014), the impact of the acoustic screen as a wind barrier is noticeable. Directly behind the barrier turbulences may form, where contaminants on suspended particles (dusts, aerosols), especially coarse ones, deposit. In the next 10–25 m, the wind velocity decreases and at a distance of 50 m is slow enough for deposition of fine fractions. The effect is not very distinct because the metal enrichment in not high, mostly because of the short time of impact (5 years). The low concentration of metals in the zone between the highway and the screen also confirms this, because this area is the most exposed for pollution additionally by runoff and splash water from the road (Werkenthin et al. 2014).

**Table 5:** The content of analysed metals and distance from the acoustic screen

	Distance	Zn	Cu	Ni	Cd	Cr	Pb
Mean	10 m^a^	27.353	6.923	7.913	0.515	24.320	1.328
	5 m	33.028	5.108	8.698	0.083	22.273	1.588
	10 m	28.860	4.533	7.953	0.363	24.458	1.358
	25 m	29.338	6.433	6.733	0.270	22.031	1.433
	50 m	29.655	6.735	8.265	0.445	23.773	1.425
	LSD_0.05_	0.074	0.125	0.103	0.315	0.020	0.073

^a^Between the screen and the highway

In the research conducted in the roadside soils by Garcia et al. (1996), highly significant correlations between the total content of cadmium, copper, zinc and lead and their bioavailable forms extracted with DTPA were noted. In this study, there did not seem to be a relationship between bioavailability and total content of the metals. Other researchers reported that lead and copper are not highly mobile metals (Pagotto et al. 2001). The study conducted by Legret and Pagotto (2006) showed that lead is the metal of high bioavailability in roadside soils. It has also been confirmed in this study—lead was the most bioavailable, mobile element—mean Pb_DTPA_ 25.79%. Due to very low total content of Pb, even such high mobility of this element is low risk for the environment. However, areas along the highways are mostly agricultural lends, focused on food production, so they should stay under especial control as to the level of pollutants. Another element of relatively high bioavailability was zinc (19.32%), whereas concentration of the forms of copper (11.35%) may lead to deficiency of the element for the plants. The rest of the analysed metals were characterised by low mobility—cadmium (9.76%), nickel (3.10%) and chromium (2.88%). Calculated correlations were significant only for bioavailable forms of Zn and clay content (*r* = −0.402; *p* < 0.05) (Fig. 2b). This negative relation indicates on crucial role of clay minerals in bonding of zinc in investigated soils. Increase of clay content leads to decrease of mobile forms of Zn.

## Conclusion

In the studied soils, natural content of analysed heavy metals was determined to be characteristic for unpolluted soils of Poland. The research confirmed low impact of the highway traffic on the content of heavy metals in soils. However, the very short period of this potential impact (5 years), the moderately polluted category of geo-accumulation index of cadmium, and high bioavailability of lead indicate the need to repeat the research within several years. Furthermore, the role of the road acoustic screen in spatial distribution of analysed metals was noticed. This barrier did not stop migration of pollutants but influenced significantly the place of deposition. Statistically the highest concentrations of the metals were found 5 and 50 m behind the screen. In comparison to other research on soils under traffic influence, the distance from the road edge where accumulation takes place is higher with acoustic screens. This is the effect of wind disturbances created by the investigated acoustic screen.
